# Supplementary material for: Association of SO2-Generating Pads before Packaging and during Cold Storage to Extend the Conservation of ‘Italia’ Table Grapes
Source: Plants (Basel). 2024 Oct 9;13(19):2827. doi: 10.3390/plants13192827 (PMC11479104; doi:10.3390/plants13192827)
Supplement: Supplementary file 1 [file plants-13-02827-s001.zip › plants-3237738-supplementary.pdf]

Table S1. Analysis of variance (means squares) for gray mold incidence (GM), mass loss (ML), shattered berries (SB), and stem browning (SBr) of 'Italia' table grapes at 30, 45, 60, 75 and 90 days of cold storage at 1.0 ± 1.0 °C, subject SO_2-generating pads before packaging and during cold storage—2020 season.

**Sources of variation**	**df**	**GM (%)**	**ML (%)**	**SB (%)**	**SBr** [a]
Treatments (T)	2	1115.81**	5.94**	4.73**	1.16**
Days (D)	4	246.55**	104.37**	10.16**	1.25**
T × D	8	66.25**	0.29	0.31**	0.02
Error A	12	4.69	0.20	0.08	0.26
Error B	48	0.97	0.16	0.01	0.01
CV-1 (%)		34.74	10.92	11.94	40.37
CV-2 (%)		15.78	9.90	4.61	9.77

df: degrees of freedom; CV: coefficient of variation; **: significant (p<0.05).
[a] Original data transformed into $\sqrt{x + 0.5}$.

Table S2. Analysis of variance (means squares) for soluble solids (SS), titratable acidity (TA), maturity index (SS/TA), and pH of the juice of 'Italia' table grapes at 30, 45, 60, 75 and 90 days of cold storage at 1.0 ± 1.0 °C, subject SO_2-generating pads before packaging and during cold storage—2020 season.

Sources of variation	df	SS	TA	SS/TA	pH
Treatments (T)	2	0.0646	5.0×10^{-5}	0.1805	0.0071
Days (D)	3	7.68**	0.0463**	24.66**	0.0723**
T × D	4	0.0617	7.0×10^{-4}	1.75	0.0041
Error A	12	0.1276	4.4×10^{-4}	1.32	0.0024
Error B	24	0.0618	0.0012	3.07	0,0078
CV-1 (%)		2.05	3.33	4.17	1.21
CV-2 (%)		1.42	5.58	6.34	2.15

df: degrees of freedom; CV: coefficient of variation; **: significant (p<0.05).

Table S3. Analysis of variance (means squares) for gray mold incidence (GM), mass loss (ML), shattered berries (SB), and stem browning (SBr) of 'Italia' table grapes at 30, 45, 60, 75 and 90 days of cold storage at 1.0 ± 1.0 °C, subject SO_2-generating pads before packaging and during cold storage—2021 season.

**Sources of variation**	**df**	**GM (%)**	**ML (%)**	**SB (%)** [a]	**SBr**
Treatments (T)	2	16.125**	0.188	2.463**	0.581
Days (D)	4	2.692**	88.211**	0.312**	6.062**
T × D	8	0.276**	0.036	0.016	0.097**
Error A	12	0.394	0.230	0.470	0.203
Error B	48	0.022	0.092	0.021	0.021
CV-1 (%)		49.40	10.10	109.52	22.1
CV-2 (%)		11.57	6.40	22.99	7.19

df: degrees of freedom; CV: coefficient of variation; **: significant (p<0.05).
[1] Original data transformed into $\sqrt{x}$.

Table S4. Analysis of variance (means squares) for soluble solids (SS), titratable acidity (TA), maturity index (SS/TA) and pH of the juice (pH) of 'Italia' table grapes at 30, 45, 60, 75 and 90 days of cold storage at 1.0 ± 1.0 °C, subject SO_2-generating pads before packaging and during cold storage—2021 season.

**Sources of variation**	**df**	**SS**	**TA**	**SS/TA**	**pH**
Treatments (T)	2	0.956	0.005	14.615	0.004
Days (D)	3	12.774**	0.048**	131.088**	0.036**
T × D	4	0.319	0.005	8.848	0.003
Error A	12	0.494	0.003	6.060	0.007
Error B	24	0.522	0.006	8.169	0.009
CV-1 (%)		4.55	8.08	11.30	2.15
CV-2 (%)		4.67	0.69	13.12	2.40

df: degrees of freedom; CV: coefficient of variation; **: significant (p<0.05).
